# Role and Dysregulation of miRNA in Patients with Parkinson’s Disease

**DOI:** 10.3390/ijms24010712

**Published:** 2022-12-31

**Authors:** Michele Salemi, Giovanna Marchese, Giuseppe Lanza, Filomena I. I. Cosentino, Maria Grazia Salluzzo, Francesca A. Schillaci, Giovanna Maria Ventola, Angela Cordella, Maria Ravo, Raffaele Ferri

**Affiliations:** 1 Oasi Research Institute-IRCCS, 94018 Troina, Italy; 2 Genomix4Life Srl, 84081 Baronissi, Italy; 3 Genome Research Center for Health-CRGS, 84081 Baronissi, Italy; 4 Department of Surgery and Medical-Surgical Specialties, University of Catania, 95123 Catania, Italy

**Keywords:** Parkinson’s disease, synucleinopathy, microRNA, miRNA, miRNA sequencing, ingenuity pathway analysis

## Abstract

Parkinson’s disease (PD) is a neurodegenerative synucleinopathy that has a not yet fully understood molecular pathomechanism behind it. The role of risk genes regulated by small non-coding RNAs, or microRNAs (miRNAs), has also been highlighted in PD, where they may influence disease progression and comorbidities. In this case-control study, we analyzed miRNAs on peripheral blood mononuclear cells by means of RNA-seq in 30 participants, with the aim of identifying miRNAs differentially expressed in PD compared to age-matched healthy controls. Additionally, we investigated the pathways influenced by differentially expressed miRNAs and assessed whether a specific pathway could potentially be associated with PD susceptibility (enrichment analyses performed using the Ingenuity Pathway Analysis tools). Overall, considering that the upregulation of miRNAs might be related with the downregulation of their messenger RNA targets, and vice versa, we found several putative targets of dysregulated miRNAs (i.e., upregulated: hsa-miR-1275, hsa-miR-23a-5p, hsa-miR-432-5p, hsa-miR-4433b-3p, and hsa-miR-4443; downregulated: hsa-miR-142-5p, hsa-miR-143-3p, hsa-miR-374a-3p, hsa-miR-542-3p, and hsa-miR-99a-5p). An inverse connection between cancer and neurodegeneration, called “inverse comorbidity”, has also been noted, showing that some genes or miRNAs may be expressed oppositely in neurodegenerative disorders and in some cancers. Therefore, it may be reasonable to consider these miRNAs as potential diagnostic markers and outcome measures.

## 1. Introduction

### microRNAs and Parkinson’s Disease

Recently, the risk genes for Parkinson’s disease (PD) have been found to be regulated by the small non-coding RNAs (sncRNA), or microRNAs (miRNAs), that may contribute to PD development through a direct regulation on the mitochondrial and immune pathways [1]. Many of these are influenced by epigenetic mechanisms, among which some are mediated by miRNAs, that regulate gene expression at a post transcriptional level by binding to their 3′ untranslated region (3′ UTR) of target messenger RNAs (mRNAs) inducing mRNA degradation and translational repression [2,3,4].

Since their discovery in 1993, miRNAs have been involved in different biological processes which maintain normal cellular life. miRNAs are a class of evolutionarily conserved non-coding RNA molecules with 18–25 nucleotides. They are transcribed by miRNA genes or detached from introns to form pri-miRNAs [5,6,7]. In nucleus, pri-miRNAs are processed by the Drosha complex to result in a 70-bp hairpin structure, called pre-miRNAs. Later, pre-miRNAs are carried into cytosol and cut edge by Dicer into 18–25 long miRNA duplexes. Mature functional microRNAs are loaded to Argonaut family protein argonaute RISC catalytic component 2 (Ago2), a major component of the RISC complex and through imperfect binding of its seed sequence to 3′ UTR of mRNA transcripts, regulate translation and stability of their targets inhibiting mRNA translation or inducing mRNA degradation [8,9].

miRNAs are widely expressed within the central nervous system (CNS) and in a tissue-specific manner, as their role is crucial for neuronal functions both during the development and in the adult brain [10]. As such, miRNAs are involved in the modulation of neuronal signaling and may enable a crosstalk between different cellular pathways, thus regulating neuronal excitability, dendritogenesis, local translation in dendritic spines, and neurotransmitter release [11,12,13]. Therefore, a disease-related downregulation of miRNA biogenesis pathway in adult neurons can modify their survival, functioning, and connectivity.

Evidence in the literature suggests that miRNA deregulation has been implicated in several neurodegenerative diseases, such as Alzheimer’s disease (AD) [14,15,16], amyotrophic lateral sclerosis (ALS) [17,18], multiple sclerosis (MS) [19], and PD [20]. Moreover, in PD, many studies over the past decade have reported dysregulation of miRNA expressions [21], and, overall, more than 15 miRNAs have demonstrated the ability to be dysregulated in the brain and/or neuronal models, cerebrospinal fluid (CSF), and blood [22]. Notably, several miRNAs targeting susceptible PD-related genes and pathways were discovered to play an important role in early pathology of PD and its progression [23,24], thus supporting their potential utility as biological markers, therapeutic targets, and outcome measures. Namely, three dysregulated miRNAs (miR-34b, miR-218, and miR-221) interact with the PD-related genes parkinsonism associated deglycase (*DJ1*), parkin RBR E3 ubiquitin protein ligase (*PRKN*), and synuclein alpha (*SNCA*) and modulate their functions in different PD cellular and animal models. In particular, the *SNCA* plays an important role in the genetics of PD inheritance [25,26,27], while others (miR-133b, miR-126, miR-132, miR-144, miR-425, and miR-124) participate in neuronal apoptosis and survival signaling pathways, and in autophagy mechanisms [22]. The following PD-specific miRNAs have been reported as potential diagnostic biomarkers in circulating fluids: miR-126, miR-144, miR-184, miR-204, and miR-221; among them, miR-144 has been proposed as an early marker. Lastly, miR-199a was proposed for stage-specific diagnosis of PD [22].

Based on epidemiological evidence that patients with some CNS disorders have a lower-than-expected probability of developing some types of cancer [28], the hypothesis of an “inverse comorbidity” relationship, driven by molecular processes common to CNS disorders and cancers that are dysregulated in opposite directions, has been proposed [29]. Namely, a transcriptomic meta-analysis of three common CNS disorders (AD, PD, and schizophrenia) and three equally common cancers (lung, prostate, and colorectal) showed a significant overlap between the genes upregulated in CNS disorders and downregulated in cancers, and between genes downregulated in CNS disorders and upregulated in cancers, and opposite expression deregulations at the level of pathways. Therefore, specific genes and pathways, the upregulation of which may increase the incidence of CNS disorders and simultaneously lower the risk of developing cancers, while the downregulation of another set of genes and pathways may contribute to a decrease in the incidence of CNS disorders while increasing the cancer risk, reinforce the concept of inverse comorbidity and reveal potential new candidates for therapy, in particular related with protein degradation processes [29].

However, despite the encouraging results and translational implications, there are several challenges and limitations in drawing definitive conclusions, mostly due to the small sample size in clinical studies, heterogeneous laboratory techniques and methodologies, and their incomplete BBB penetrance. Therefore, developing an optimal delivery system and unravelling druggable targets of miRNAs in both experimental and human models and clinical validation of the results may pave way for novel therapeutics in PD.

In this study, a systematic analysis of miRNA, followed by functional analysis of the results, was performed to evaluate their possible deregulation in PD patients and to fully explore the role played by miRNAs in regulating various genes responsible for the onset and progression of PD. In addition, we investigated whether specific miRNAs are able to target genes and, thus, modulate their functions in PD patients. As such, targeting specific miRNAs may be potentially used for diagnostic and prognostic purposes.

## 2. Results

### 2.1. Characterization of Small Non Coding RNAs Deregulation in PD Patients

Small RNA expression profiling was performed by next-generation sequencing in PD patients and controls, after filtering out low-quality reads and trimming the adaptors. The high-quality reads obtained were aligned against the human genome reference.

**Figure 1:**
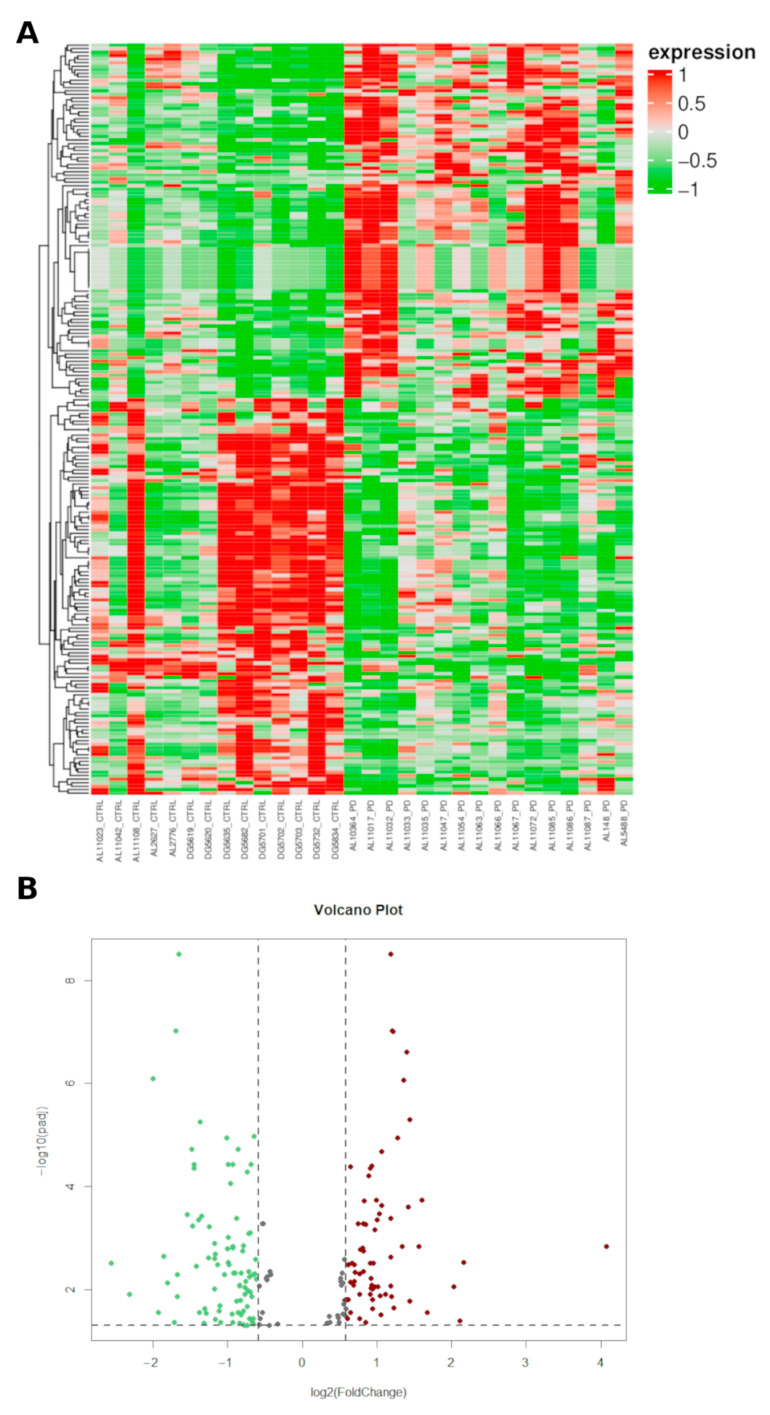
miRNA profiling. (**A**) Heatmap of the supervised hierarchical clustering analysis on the expression profiles of the of differentially miRNAs (padj ≤ 0.05 and |FC| ≥ 1.5) in patients with Parkinson’s disease compared to healthy controls. The Heatmap was built through ComplexHeatmap R package setting clustering distance = “euclidean”, and clustering method = “complete”. Expression values lower or higher than the median are shown in green or red, respectively (**B**) Volcano plots of DEmiRNAs (padj ≤ 0.05 and |FC| ≥ 1.5). The gray arrows indicate points-of-interest that display both large log2 fold-changes (*x* axis) and high statistical significance (−log10 of padj, *y* axis). Red: overexpressed miRNAs; green: downexpressed miRNAs (Supplementary Table S1); downexpressed miRNAs: Supplementary Table S1.

To investigate the overall miRNA expression differences between the cohorts, hierarchical clustering analyses were performed. PD samples showed expression heterogeneity, as some of these subjects clustered close to/among controls. This suggests a molecular diversity in PD cases that is reflected by miRNA expression. More in details, we identified 1752 unique miRNAs, of which 179 unique miRNAs differentially expressed between the two groups (PD vs. controls). Among them, 67 unique miRNAs were significantly (padj ≤ 0.05 and |FC| ≥ 1.5) upregulated and 85 unique miRNAs (padj ≤ 0.05 and |FC| ≤ −1.5) were significantly downregulated in PD patients compared to controls (Figure 1, Supplementary Table S1 and Supplementary Figure S1). The normalized count of miRNAs is available at ArrayExpress (E-MTAB-12087).

**Figure 2:**
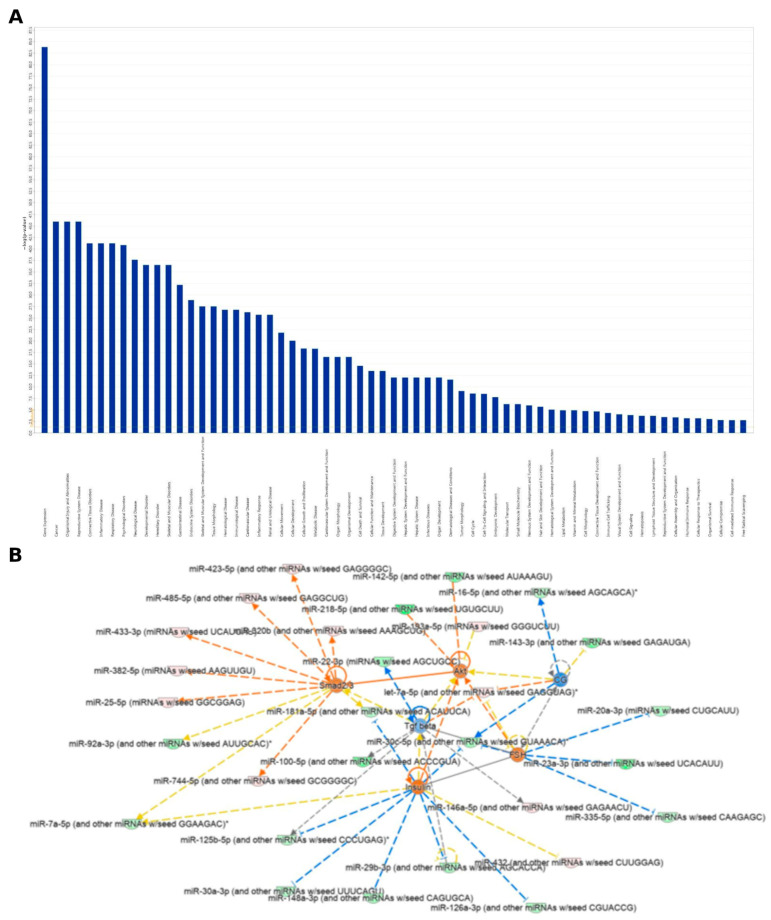
Ingenuity Pathway Analysis (IPA). (**A**) Diseases and biological functions enriched by the IPA “Core Analysis” for the miRNAs differentially (padj ≤ 0.05 and |FC| ≥ 1.5) expressed between controls and patients with Parkinson’s disease. (**B**) IPA network of some miRNAs expressed displayed as nodes and edges (biological relationship between nodes). The color intensity of each node represents fold change expression, i.e., red (upregulated) and green (downregulated). The edges connecting the miRNAs/genes to the respective functions represent the predicted relationships, blue representing inhibition and grey representing effect not predicted based on the IPA activation z-scores, combination of directional information encoded by the gene expression with information curated from the literature (Supplementary Table S3).

In order to investigate the pathways affected by differentially expressed miRNAs (DEmiRNAs) and assess whether some pathways might be potentially associated with PD susceptibility, we performed an enrichment analysis by using the Ingenuity Pathway Analysis (IPA) tools. IPA pathway analysis revealed that the DEmiRNAs were mainly enriched into different Diseases and biological Functions (padj ≤ 0.05 and |FC| ≥ 1.5, Figure 2A). In particular, one of the more enriched categories was Neurological Disease with several Diseases or Functions Annotation (e.g., cerebral disorder, AD, PD, neuroblastoma), and with a significant Benjamini-Hochberg *p* value (B-H *p*-value) (Supplementary Table S2). As shown in Figure 2B by the DEmiRNAs IPA Network Analysis, we identified several nodes and interaction with high score. Among these, we highlight the SMAD 2/3 signaling pathways directly targeted by two up-regulated (miR-1275 and miR-432_sp) and one downregulated miRNAs (miR-99a_sp).

Gene-disease associations analysis performed by DisGeNET platform highlight the role of SMAD 3 in several neurodegenerative process included neuroinflammation processes closely related to etiopathogenesis of PD. Moreover, SMAD3 signaling has been shown to play a crucial role in the development and homeostasis of microglia regulating microglial activity, contributing to the maintenance of the neural environment. Microglia is involved in nigrostriatal dopaminergic neurodegeneration by releasing proinflammatory factors (Supplementary Table S3).

### 2.2. Analysis of the Differentially Expressed miRNAs’ Target Genes

We predicted DEmiRNAs target genes using the miRWalk software, a tool for automating miRNA-targeting gene analysis procedures. This analysis was performed considering experimentally verified miRNA-target interactions with score 0.95 on 3′UTR. Using this method, we obtained for the 67 upregulated miRNAs approximately 450,509 targets, of which 17,176 unique target genes. Instead for 85 downregulated miRNAs, about 125,606 targets were found, of which 14,071 unique target genes, and 13,759 in common between the groups (Supplementary Table S4). Considering that miRNA upregulation could be related to downregulation of its mRNA targets, and vice versa, we investigated a possible dynamic inverse connection between miRNA and mRNA levels. To evaluate this possibility, we first explored for the attendance of perfect or imperfect matches between the seed sequence of each regulated miRNA and the UTR of their putative mRNA targets.

**Figure 3:**
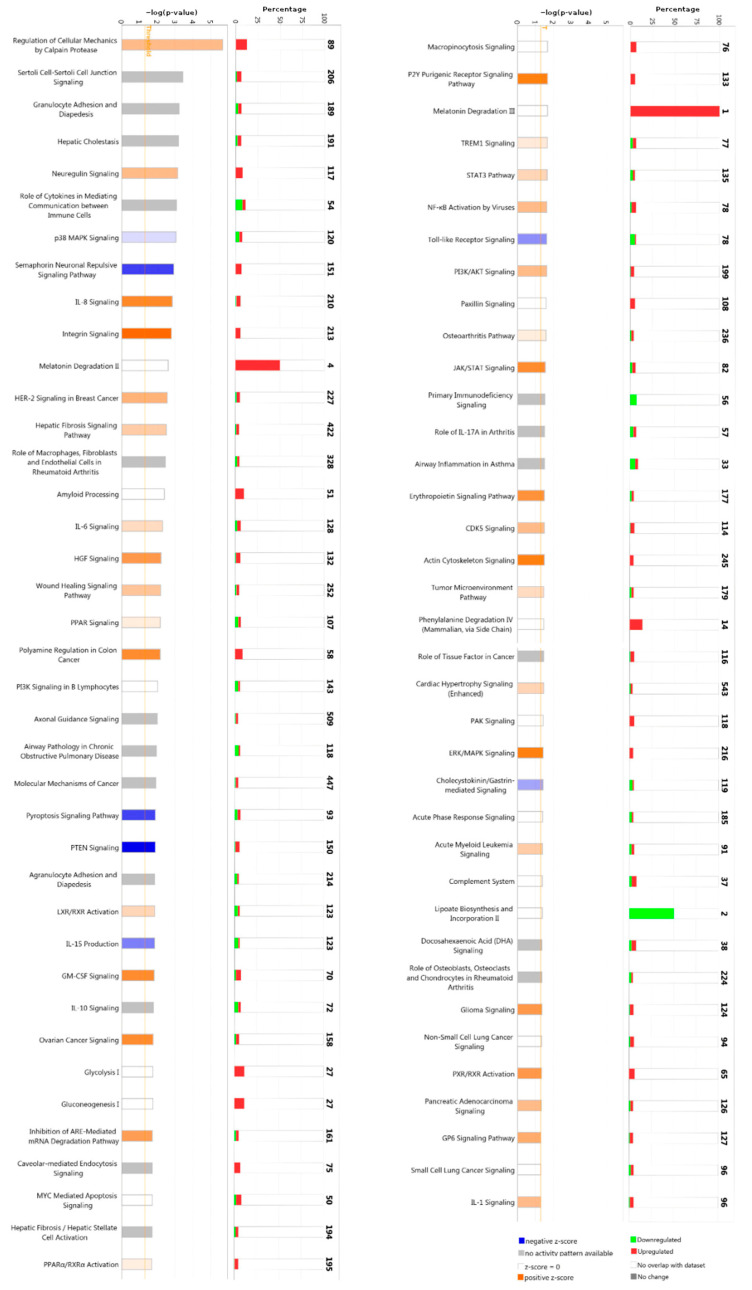
Canonical Pathway Ingenuity Pathway Analysis (IPA) on miRNA target genes. The Canonical Pathway Analysis in the IPA associates probe sets with the canonical pathways in the Ingenuity’s Knowledge Base to identify gene sets involved with a statistical significance (left column). Number of over- and underexpressed genes involved in each enriched pathway are show in the right column.

In order to mainly focus on the most interesting target-genes, we considered for the next IPA pathway analysis (Figure 3) only the putative mRNA targets that we found statistically significant by mRNA expression profiling in the same previously published patient cohort (|FC (PD vs. CTRL)| ≥ 1.5 and padj ≤ 0.05) [25]. We found 497 genes as significant targets of all DEmiRNAs (Supplementary Table S5).

**Figure 4:**
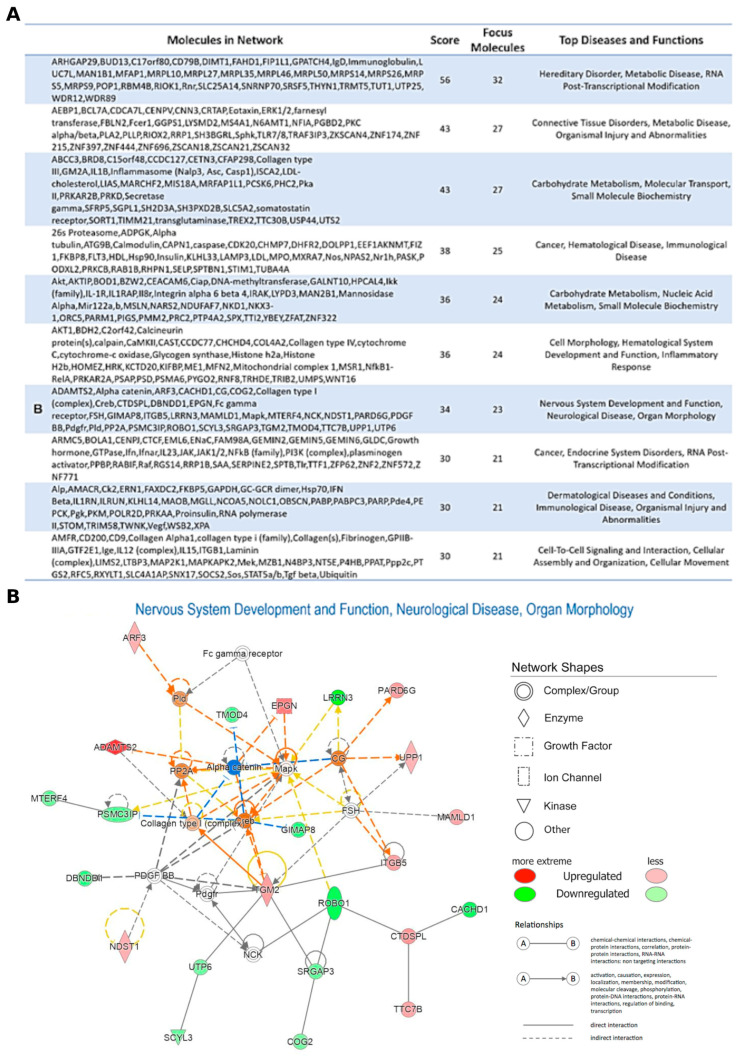
Network Ingenuity Pathway Analysis (IPA). (**A**) Top 10 Networks obtained by IPA Core Analysis. (**B**) Nervous System Development and Function, Neurological Disease, Organ Morphology Network. Red: upregulated transcripts; green: downregulated transcripts.

**Figure 5:**
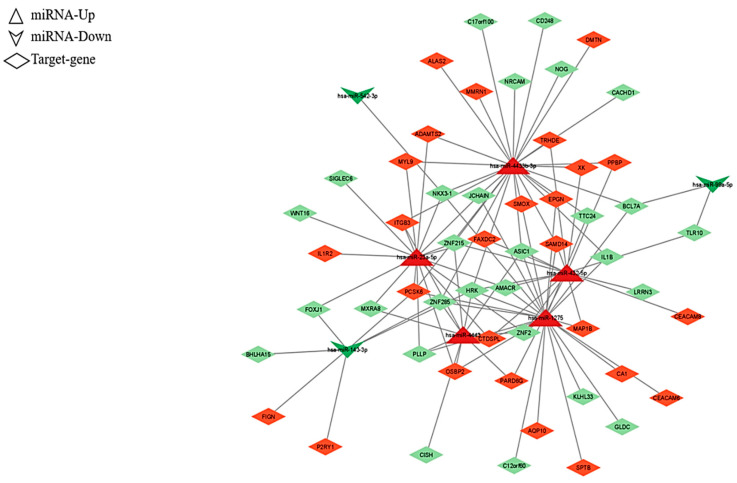
miRNA-target gene network. The network is based on the dysregulated miRNAs and their target genes. The green color represents downregulated miRNAs while the red color represents upregulated miRNAs (Supplementary Table S7).

Interestingly, among the top five canonical pathway enriched, Neuregulin signaling (117 upregulated target genes) was found (Figure 3, right column). Moreover, in order to obtain an integrative view of interactions between the dysregulated miRNAs and their targets, functional network analysis was performed, which revealed that the candidate genes were associated with several biological functions, such as binding, molecular function, and catalytic activity (Figure 4). Regulatory networks of miRNA–mRNA interactions were constructed and visualized using the Cytoscape software version 3.8.0 (The Cytoscape Consortium, http://www.cytoscape.org/). In particular, we deepened on edges of all up- and downregulated miRNAs–target connected and then we focused on miRNA–mRNA targets regulatory network filtered by fold-change (|FC| ≥ 1.5). This analysis led to the identification of 108 interactions for miRNA-nodes, involving 26 Up and 28 down-targets. (Figure 5 and Supplementary Table S6). These miRNAs-mRNAs genes show connections with other genes and may play an important role in the network. These genes are targeted by a group of miRNAs, suggesting that they also participate in the network of PD.

We focused our attention on top five upregulated (hsa-miR-1275, hsa-miR-23a-5p, hsa-miR-432-5p, hsa-miR-4433b-3p, and hsa-miR-4443; Figure 4A) and downregulated DEmiRNAs (hsa-miR-142-5p, hsa-miR-143-3p, hsa-miR-374a-3p, hsa-miR-542-3p, and hsa-miR-99a-5p; Figure 4B), highest statistically significant, filtered by |FC| ≥ 2.5, and BASE MEAN (mean value of the normalized expression value in PD and controls). In this way, looking for the top five upregulated miRNAs, we found 338 target RNAs expressed, of which, 27 were downregulated (FC ≤ −1.5) and 24 were upregulated (FC ≥ 1.5), and, for the 5 downregulated miRNAs, we found 60 significant genes filtered for padj of which seven downregulated (FC ≤ −1.5) and three upregulated (FC ≥ 1.5) (Supplementary Table S7).

**Figure 6:**
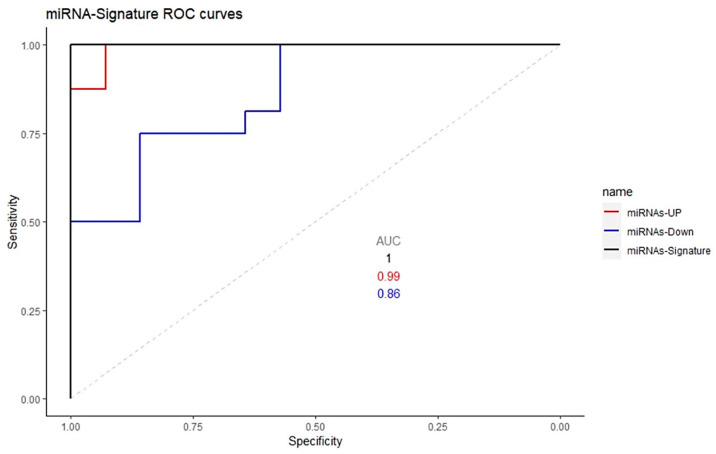
Receiver operating characteristic (ROC) curve analysis of 5 upregulated and downregulated miRNA.

To evaluate the power of diagnostic value of this molecular signature built on selected miRNAs (hsa-miR-1275, hsa-miR-23a-5p, hsa-miR-432-5p, hsa-miR-4433b-3p, hsa-miR-4443), and (hsa-miR-142-5p, hsa-miR-143-3p, hsa-miR-374a-3p, hsa-miR-542-3p, hsa-miR-99a-5p), a receiver operating characteristic (ROC) analysis was performed. The ROC curves of the five most up- and downregulated miRNAs revealed combined area under the curve (AUC) values of 0.99 and 0.88, respectively (Figure 6), indicating their potential value as biomarkers.

## 3. Discussion

The main finding of this study is that the biological functions of differentially expressed miRNAs appear to be closely related to gene expression, and the role of miRNAs involved in cancer, which could be partly explained by an “inverse comorbidity” mechanism, as recently proposed [30]. Indeed, the recent literature has shown an inverse correlation between cancer and neurodegeneration [31], often based on inverse comorbidity phenomena, thus demonstrating how specific genes or miRNAs may be oppositely expressed in neurodegenerative diseases and in some cancers [32,33,34]. In addition, many of the differentially expressed miRNAs are linked to numerous inflammatory, neurological, and muscular diseases (Figure 2A), all of which may be related to the pathogenesis and pathophysiology of PD. Among them, the Transforming Growth Factor-beta (TGF-β) signaling pathway plays a central role in tumorigenesis [35,36,37] and participates in the proliferation, migration, and invasion of various cancer cells [38]. We found that the TGF-β signaling pathway is inhibited (Figure 2B) and since, in general, proliferative processes are inhibited in neurodegenerative diseases (including PD), our findings might support and further elucidate the neurobiological processes underlying PD through the role of some differentially expressed miRNAs.

As shown in Figure 3, the Canonical Pathway IPA on miRNA target genes, which associates probe sets with canonical pathways from the Ingenuity Knowledge Base to identify the sets of genes involved in each enriched pathway, revealed other interesting results. Among them, the “Melatonin II Degradation” and the “Melatonin III Degradation” pathways showed significant overexpression. This over-regulation, however, affects the regulation of 50% to 100% of all genes involved in these pathways. Indeed, the role of melatonin as a chronobiotic and cytoprotective agent for PD has been clearly established [39]. In Parkinsonian patients, circulating melatonin levels are consistently altered, and the potential therapeutic value of melatonin on certain sleep disorders in PD, such as the REM sleep behavior disorder (RBD, which may precede the onset of motor symptoms in PD by years and is an index of worse prognosis [40]) has been examined. The low density levels of melatonin receptors 1A and 1B in the substantia nigra and amygdala found in PD patients supports the hypothesis that the sleep/wake cycle alteration found in PD may be due to an alteration of the melatoninergic system [41]. Accordingly, daily administration of 3–12 mg of melatonin at bedtime has been shown to be effective in the treatment of RDB and may slow down the neurodegeneration underlying PD. The possible mechanisms include: inhibition of certain pathways related to apoptosis, autophagy, oxidative stress, inflammation, α-synuclein aggregation, and dopamine leakage, among others [42,43]. As a whole, this finding might explain the possibility of an abnormally accelerated catabolism of melatonin in these patients, which eventually leads to worsen, at least in part, the neurodegenerative process underlying PD.

A lower amount of evidence is available for another pathway that seems to be clearly involved in PD, i.e., lipoate biosynthesis and incorporation. The two genes involved by dysregulated miRNAs that we found in PD patients were downregulated and account for 50% of all genes involved in this pathway. This downregulation, which essentially weakens the mechanisms of cellular defense, could contribute to PD-related neurodegeneration based on the known antioxidant and neuroprotective effect of lipoic acid in PD. Specifically, both lipoic acid and its reduced form, dihydrolipoic acid, act against reactive oxygen species, reducing oxidative stress [44]. More recently, alpha-lipoate has been shown to attenuate neurotoxicity induced in primary astrocytes and to changes in gene expression that could be caused by toxins in primary astrocytes, probably through oxidative stress and excitotoxicity, culminating in cell death [45].

Finally, among all the other pathways included, the one related to the regulation of cell mechanics by the protease calpain might also play a role, although the relatively high amount of differentially expressed genes involved in the dysregulation of miRNAs (89) accounts for about 15% of all genes involved in this pathway. However, the high statistical significance observed and the fact that all these genes are downregulated in PD clearly support the role of calpain activation and progression of inflammatory cycles. Calpain, a neutral protease, is a regulator of various cells, including T cells, microglia, and astrocytes, leading to persistent neuroinflammatory responses and neuronal loss [46]. In this context, calpain plays a central role in the cleavage and aggregation of toxic α-synuclein [47]. Therefore, calpain inhibitors have been proposed as potential therapies for PD to prevent calpain-related inflammation and neurodegenerative responses [48].

**Figure 7:**
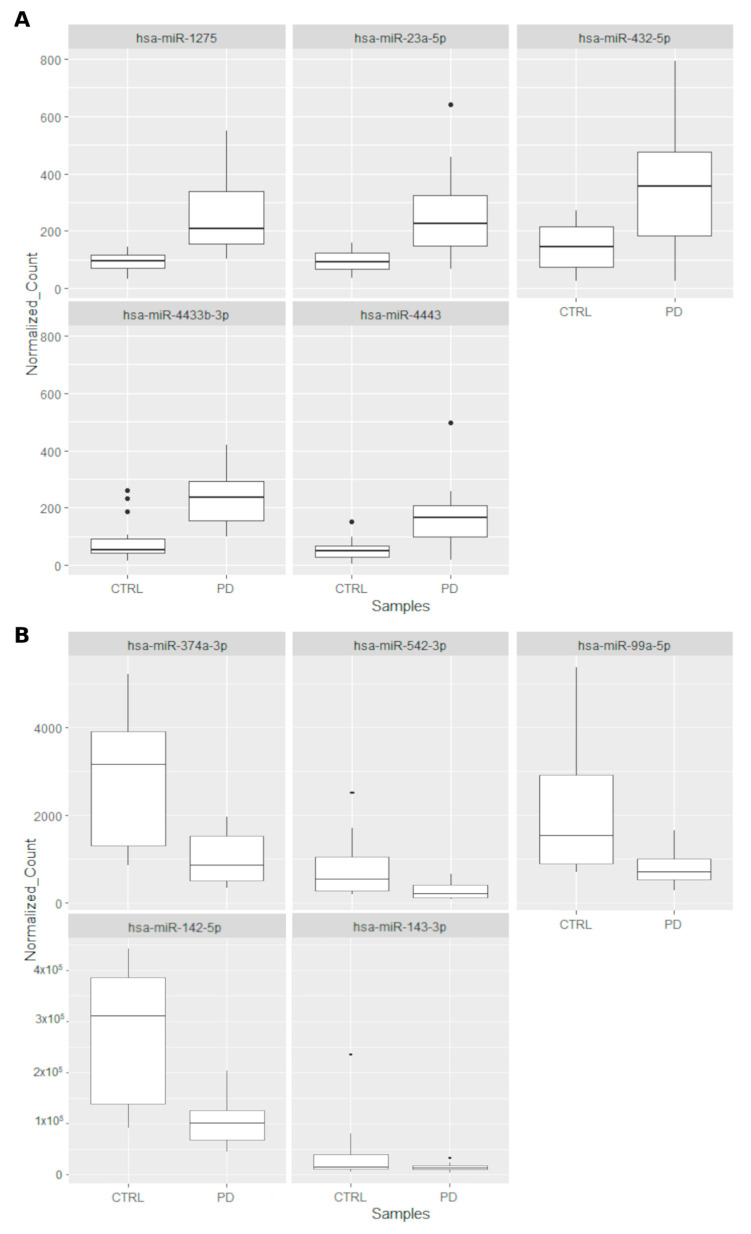
BoxPlots showing the expression level (Normalize Count—Supplementary Table S1) of top five upregulated miRNAs (**A**) and top 5 Down regulated miRNA in PD vs. controls (|FC| ≥ 2.5 and padj ≤ 0.05) (**B**). Rectangles define 25th and 75th percentiles, horizontal lines show median values, whiskers indicate extreme values, *t*-test *p* < 0.05).

In this study, the hsa-miR-1275, hsa-miR-23a-5p, hsa-miR-432-5p, hsa-miR-4433b-3p, and hsa-miR-4443 were found to be upregulated (Figure 7A), whereas the hsa-miR-142-5p, hsa-miR-143-3p, hsa-miR-374a-3p, hsa-miR-542-3p, and hsa-miR-99a-5p were downregulated (Figure 7B). As summarized in the Supplementary Table S1, although all of these miRNAs have been involved in a number of neurological, neuropsychiatric, and neuro-oncological disorders; to date, only few of them have been associated with preclinical or human models of PD.

Namely, the miRNA expressing profiles in the A53T mutant alpha-synuclein transgenic mice and PD patients [49] revealed that 11 miRNAs were differently expressed in the CSF, including the hsa-miR-542-3p, which showed accuracy for the prediction of PD. Moreover, the ordered logistic regression analysis showed that the severity of PD strongly correlates with the expression of some miRNAs, thus suggesting then as potential biomarkers for PD [49]. More recently, the hsa-miR-142-5p was found to be downregulated in mouse and cell models of PD, along with the finding that miR-142-5p inhibition abated the neuroprotective effect of berberine [50]. Moreover, this further supports the role of hsa-miR-142-5p in PD pathogenesis, being this miRNA a neuroprotective regulator via the autophagy-related influence cell viability, eventually leading to neuronal death [51]. Accordingly, the hsa-miR-142-5p inhibition promotes the pathomechanisms underlying neurodegeneration [50,51]. Notably, high levels of hsa-miR-142-5p were also found in cervical carcinoma, where it is implicated in tumorigenesis and constitutes a therapeutic target [52].

Aside from these cases, however, to date, none of the other miRNAs we found to be differentially expressed in these patients has been associated with PD. Nevertheless, an association with a number of different neurological, neuropsychiatric, or neuro-oncological disorders has been reported (Supplementary Table S1), and their clear role in several tumors not primarily affecting the central nervous system, e.g., the hsa-miR-1275 in women with breast cancer; indeed, the has-miR-1275 has been proposed as a biomarker for the early diagnosis and treatment of this cancer [53]. It should be noted that other studies have highlighted the role of downregulated hsa-miR-1275 in glioblastoma, a rare but highly malignant astrocytic tumor of the neuroepithelial tissue, which is characterized by a survival time of less than one year in most affected patients [54]. Conversely, the same miRNA was found to be significantly increased in human epilepsy of unknown etiology, thus hypothesizing a model of inverse comorbidity between these two disorders [55].

Another relevant example derived from our results comes from the hsa-miR-23a-5p, which is known to be underexpressed in cervical carcinoma, a malignant cancer with a high incidence and recurrence rate [56]. Notably, downregulation of this miRNA has been found in subjects with glioblastoma compared with non-tumor tissues [57], whereas it is upregulated in subjects with epilepsy [58] or schizophrenia [59].

Similarly, the hsa-miR-432-5p is strongly underexpressed in colorectal cancer cells and correlated with some clinical-pathological factors, such as invasion, lymph node metastasis, and tumor node metastasis [60]. The same holds true for liver carcinoma, in which an underexpression of hsa-miR-432-5p was noted [61]. However, the same miRNA appears to be overexpressed in subjects with autism [62] and in those with MS [63].

A study of circulating miRNAs as potential diagnostic biomarkers of poor sleep quality revealed that has-miR-4433b-3p might exert a role. Accordingly, low sleep quality was associated with lower expression levels of hsa-miR-4433b-3p compared to the control group [64]. Conversely, when overexpressed, this miRNA has been identified as a potential biomarker of breast cancer [65].

The hsa-mir-4443 was reported to be underexpressed in head and neck squamous cell carcinoma [66], and in osteosarcoma and hepatocellular carcinoma, whereas, when inhibited, it might suppress cell migration, invasion, and proliferation [67,68]. In contrast, the hsa-mir-4433 has been shown to be overexpressed in the cerebral cortex of subjects with AD or Huntington’s disease [69]. Similarly, the has-miR-374a-3p is upregulated in subjects with colorectal cancer compared to non-cancerous tissues [70] and in bladder carcinoma [71]. However, the hsa-miR-374a-3p was shown to be dysregulated in subjects with ischemic stroke, thus suggesting a role of candidate biomarker [72].

Among brain tumors, an in silico analysis study identified the hsa-miR-542-3p as overexpressed in glioblastoma multiforme, further identifying it as a biomarker [73]. Conversely, different studies have found it underexpressed in neuroblastoma, being its overexpression possibly protective towards this tumor [74,75,76,77]. The hsa-miR-542-3p seems to behave similarly in astrocytoma, in which it is downregulated in cell lines and tissues [78].

The exosome has-miR-99a-5p is detected at elevated levels in the serum of women with epithelial ovarian cancer compared to healthy controls or with benign tumors [79]. However, this miRNA expression was found to be low in squamous cell head and neck cancer and cervical cancer [80]. Moreover, the manipulation of the has-miR-99a-5p, leading to its overexpression, results in apoptosis and glycolysis arrest in cervical carcinoma cells [81]. It is worth to mention that, among others, this miRNA can be particularly used as a CSF diagnostic marker for various neurological diseases [82], although further investigation is warranted before systematic clinical translation.

An intriguing role seems to be played by the hsa-miR-143-3p, which is upregulated in brain metastases of lung cancer, which are the leading cause of mortality in these patients, dramatically reducing overall survival to no more than one year. In these patients, the hsa-miR-143-3p expression confers metastatic potential to tumor cells, increases their angiogenic capabilities, and supports tubulin depolymerization [83]. In addition, the hsa-miR-143-3p is upregulated in women with ovarian cancer [84]. Simultaneously, however, the hsa-miR-143-3p decreases the phosphorylation of the amyloid precursor protein, thus lowering the levels of amyloid-β40 and amyloid-β42. Therefore, it is not surprising that the hsa-miR-143-3p is downregulated in the hippocampus of AD patients [85].

Overall, the hsa-miR-1275, hsa-miR-23a-5p, hsa-miR-432-5p, and hsa-miR-4443, which were all overexpressed in our PD patients, are underexpressed in many tumors, including glioblastoma. Similarly, the underexpressed miRNAs observed in this study (hsa-miR-142-5p, hsa-miR-143-3p, hsa-miR-374a-3p, hsa-miR-542-3p, and hsa-miR-99a-5p) are overexpressed in various tumors, especially in glioblastoma (Table 1). Taken together, this supports the role of dysregulated miRNAs in PD and agrees with the hypothesis of inverse comorbidity between cancers and neurodegenerative diseases.

Lastly, as shown in Figure 4A, leucine rich repeat neuronal 3 *(LRRN3)* is a target gene of the hsa-miR-432-5p, that we found upregulated (Supplementary Table S2). As a consequence, this miRNA may play an inhibitory role on the target gene *LRRN3*, thus confirming previous findings in PD [86,87]. Additionally, the hsa-miR-4433b-3p, which was found to be overexpressed, targets the noggin gene *(NOG)*, that is underexpressed in PD [87]. Therefore, the expression of *LRRN3* and *NOG* genes, both clearly involved in the neurological network of PD (Figure 4) might be useful to identify them as potential biomarkers. A similar approach might be proposed for the forkhead box J1 gene (*FOXJ1)* and related hsa-miR-23a-5p, which is involved in immune and inflammatory disorders [88,89]. Accordingly, its overexpression was identified in ALS [90] and, although its expression in PD and its relationship with ALS are still debated, it is clearly involved in neurodegeneration.

Lastly, the *PCSK6* (alternative label of *PACE4*) was found to be underexpressed in PD [87], along with the downregulation of the hsa-miR-143-3p observed in this study (Figure 6; Supplementary Table S2). In this context, it is known that in primary human melanoma and its metastases, *PACE4* expression is specifically detected (except for the case of melanoma in situ), thus indicating that *PACE4* might be a regulator of melanoma cell aggressiveness [91]. Epidemiologically, PD patients are known to be at reduced risk of several tumors (especially colon, rectal, colorectal, and lung cancer), but at increased risk of brain cancer and melanoma, thus confirming the mechanism of “inverse comorbidity” between PD and cancers [87] with the exception of brain cancer and melanoma.

## 4. Materials and Methods

### 4.1. Participants

This case-control study consists of a miRNA analysis on peripheral blood mononuclear cells (PBMCs) performed by RNA-seq in 30 participants of Sicilian ancestry: 16 PD patients and 14 age- and sex-matched healthy controls, all recruited at the Oasi Research Institute-IRCCS of Troina (Italy). PD patients (10 males and 6 females, mean age 68.00 ± 6.47 years; mean disease duration 5.75 ± 3.80 years) were diagnosed according to the latest diagnostic criteria for PD [92]. Overall, five patients exhibited an akinetic-rigid phenotype and two were tremor-dominant, whereas the remaining subjects (nine) showed mixed features. Controls (10 males and 4 females, mean age 71.94 ± 13.19 years) were drug-free, did not have any history of neurological or psychiatric disorders, and their neurological exam was entirely normal. A summary of the clinical-demographic characteristics of PD patients, along with their main comorbidities and drug(s) taken is reported in the Supplementary Table S8. It should be acknowledged that these patients came from a clinical environment and, therefore, a degree of clinical heterogeneity was unavoidable. Accordingly, some heterogeneity in disease duration and sleep disorders was noted, but substantially not in other clinical features, such as PD phenotype and comorbid conditions. Moreover, to overcome any heterogeneity from different therapies, the total daily levodopa equivalent dose was calculated and provided for each patient.

Informed consent was obtained from patients and controls or, when necessary, from their relatives. The study was conducted in accordance with the Declaration of Helsinki of 1964 and its later amendments, and the Ethics Committee of the Oasi Research Institute-IRCCS of Troina (Italy) approved the protocol on 4 May 2021 (2021/05/04/CE-IRCCS-OASI/43).

### 4.2. miRNA Extraction

PBMCs separation was performed using Ficoll-Paque (Ficoll Plaque PLUS–GE Healthcare Life Sciences, Piscataway, NJ, USA. Total RNA extraction from PBMC was performed using TRIzol reagent (TRIzol Reagent, Invitrogen Life Technologies, Carlsbad, CA, USA), according to the manufacturer’s instructions. RNA was stored at −80 °C until further processing. RNA concentration and purity were evaluated using NanoDrop™ 2000/2000c (Thermo Fisher Scientific, Waltham, MA, USA), whereas sample integrity was analyzed by TapeStation 4200 (Agilent Technologies, Santa Clara, CA, USA) using RNA ScreenTape Assay 2.7.

### 4.3. miRNA Sequencing and Data Analysis

An amount of 500 ng purified RNA was used for sequencing library preparation using NEXTFLEX Small RNA-Seq Kit v3 (Perki-nElmer, Waltham, MA, USA) according to the manufacturer’s instructions. Libraries were quantified using the TapeStation 4200 (Agilent Technologies) and Qubit fluorometer (Invitrogen Co.), then pooled such that each index-tagged sample was present in equimolar amounts. The pooled samples were subject to cluster generation and sequencing using an Illumina NextSeq 550 Dx System (Illumina, Santa Clara, CA, USA) in a 1 × 75 single-end format. Fastq underwent to Quality Control using FastQC tool http://www.bioinformatics.babraham.ac.uk/projects/fastqc.

The tool sRNAbench [93] was used to remove adapter sequences based on the kit used, the low quality reads, to obtain the miRNA expression profiling with respect to miRBase [94] database (version22-GRCh38-GCA_000001405.15). Reads that had a sum read count less than 10 were excluded, in all samples used. To identify differentially expressed miRNA in PD vs. controls, the DESeq2 [95] (version 1.36.0) algorithm was used.

miRNAs expression was considered as significantly differentially expressed between controls and patients with PD when it was characterized by |Fold-Change| (|FC|) ≥ 1.5 (*p* ≤ 0.05). miRNA target prediction was performed using miRWalk [96] v.3.0, which calculates a score for each putative miRNA-mRNA interaction. To identify highly predicted mRNA targets we considered only miRNAs characterized by a prediction score equal to 1 and identified by at least two out of three algorithms present in miRWalk database. Differentially expressed mRNAs with |FC| ≥ 1.5 were considered as putative miRNA targets, considering both experimentally validated and highly predicted interactions.

Deregulated canonical pathways and functions analysis was conducted by Ingenuity Pathway Software (IPA, Ingenuity System). Data integration, heatmaps demonstrating differentially expressed miRNAs, and functional enrichment plots were prepared using the R/Bioconductor packages.

The raw data (.fastq files) and the normalized count of miRNAs identified are available at ArrayExpress (E-MTAB-12087).

## 5. Conclusions

We found a dysregulation of the miRNAs hsa-miR-1275, hsa-miR-23a-5p, hsa-miR-432-5p, hsa-miR-4433b-3p, hsa-miR-4443, hsa-miR-142-5p, hsa-miR-143-3p, hsa-miR-374a-3p, hsa-miR-542-3p, and hsa-miR-99a-5p; almost all of these miRNAs were found to be dysregulated for the first time in a study involving patients with PD. Future studies will be needed to test the possibility to use them as diagnostic biomarkers for this α-synucleinopathy, and, in particular, to assess their eventual differential expression within this category of neurodegenerative conditions. Of note, the TGF-β signaling pathway was found to be inhibited, possibly indicating an inhibition of the proliferative processes that are typical of tumors. Additionally, the significant overexpression of “melatonin II degradation” and “melatonin III degradation” pathways seem to correlate with the well-known sleep disorders that PD subjects may exhibit early among the non-motor symptoms of this disease. Finally, our results further support the concept of “inverse comorbidity” between PD and some cancers, which involves several miRNAs that were found to be dysregulated in the present study.

## Figures and Tables

**Table 1:** List of differentially expressed miRNAs found in this study and their association with Parkinson’s disease (PD) or other neurological, neuropsychiatric, or neuro-oncological disorders.

Level of Expression Found	miRNA	Association with PD Models or Patients (References)	Association with Other Neurological, Neuropsychiatric, or Neuro-Oncological Disorders (References)
Up	miR-1275	No	Multiple sclerosis [PMID: 35273684]; Human epilepsy of unknown etiology [PMID: 33251277]; Atypical meningioma [PMID: 32426270]; Ischemic stroke [PMID: 31935511]; Glioblastoma [PMID: 31162799; PMID: 25129238; PMID: 22736761]; Neural tube defects [PMID: 22642222]; Atherosclerosis [PMID: 31935511; PMID: 31506771]
Up	miR-4433b-3p	No	Poor sleep quality [PMID: 34234603]; Prediction for stroke [PMID: 31136284]
Up	miR-23a-5p	No	Cerebral ischemia in mice [PMID: 35547763]; Glioblastoma [PMID: 32783743]; Multiple sclerosis [PMID: 32432792]; Atherosclerosis [PMID: 30227118]; Epilepsy [PMID: 26382856]; Schizophrenia and schizoaffective disorder [PMID: 26173148]
Up	miR-4443	No	Glioblastoma [PMID: 35992881; PMID: 35300350; PMID: 29678219; PMID: 29643013]; Alzheimer’s disease and Huntington’s disease [PMID: 35929619]; Sporadic amyotrophic lateral sclerosis [PMID: 34776863]; Stroke-induced immunosuppression [PMID: 32337817]
Up	miR-432-5p	No	Autism [PMID: 35342389]; Glioma [PMID: 33915163; PMID: 33220929; PMID: 32629066; PMID: 31504797; PMID: 31246330]; Neuroblastoma [PMID: 33837793; PMID: 25762502]; Multiple sclerosis [PMID: 31663645; PMID: 29084979]; Schizophrenia [PMID: 31297041]
Down	miR-374a-3p	No	Ischemic stroke [PMID: 34456585]
Down	miR-542-3p	Yes [PMID: 27965467]	Neuroblastoma [PMID: 33987474; PMID: 32412051; PMID: 32021273; PMID: 25046253]; Glioma [PMID: 33922649]; Glioblastoma [PMID: 30816530]; Astrocytoma [PMID: 26286747]; Cerebral infarction prevention [PMID: 33407827]; Ischemic stroke [PMID: 27151415]; Epilepsy [PMID: 31702493]; Neointima formation [PMID: 26026397]
Down	miR-142-5p	Yes [PMID: 34427876] Yes [PMID: 32127787]	Spinal muscular atrophy [PMID: 35584175]; Intracranial germ cell tumors [PMID: 35171328]; Cognitive impairment [PMID: 34302879]; Epilepsy [PMID: 33041753; PMID: 32800995; PMID: 32439493; PMID: 24454901]; Alzheimer’s disease and other dementia [PMID: 32251633; PMID: 26981236]; Glioma [PMID: 32238705]; Multiple sclerosis [PMID: 30175165; PMID: 28302134; PMID: 28114622]
Down	miR-143-3p	No	Acute ischemia/stroke [PMID: 35571371; PMID: 35401659; PMID: 33038923; PMID: 28724745]; Alzheimer’s disease [PMID: 34775974; PMID: 32337953]; Rare muscular dystrophy [PMID: 35393236]; Epilepsy [PMID: 35055144]; Amyotrophic lateral sclerosis [PMID: 34454204]; Intracranial Aneurysmal Tissues [PMID: 34185228]; Atherosclerosis [PMID: 33603842]; Peripheral nerve tumors [PMID: 32076030]; Multiple Sclerosis [PMID: 28114622]
Down	miR-99a-5p	No	Cerebro-spinal fluid biomarkers [PMID: 28983117]; Ischemic stroke [PMID: 30276300]; Neurotoxicity to PM2.5 [PMID: 27539004]

## Data Availability

The raw data and the normalized count of miRNAs identified are available at ArrayExpress (E-MTAB-12087).

## Supplementary Materials

The supporting information can be downloaded at: https://www.mdpi.com/article/10.3390/ijms24010712/s1.
