# Supplementary material for: Role and Dysregulation of miRNA in Patients with Parkinson’s Disease
Source: Int J Mol Sci. 2022 Dec 31;24(1):712. doi: 10.3390/ijms24010712 (PMC9820759; doi:10.3390/ijms24010712)
Supplement: Supplementary file 1 [file ijms-24-00712-s001.zip › Supplementary_Figure S1.pdf]

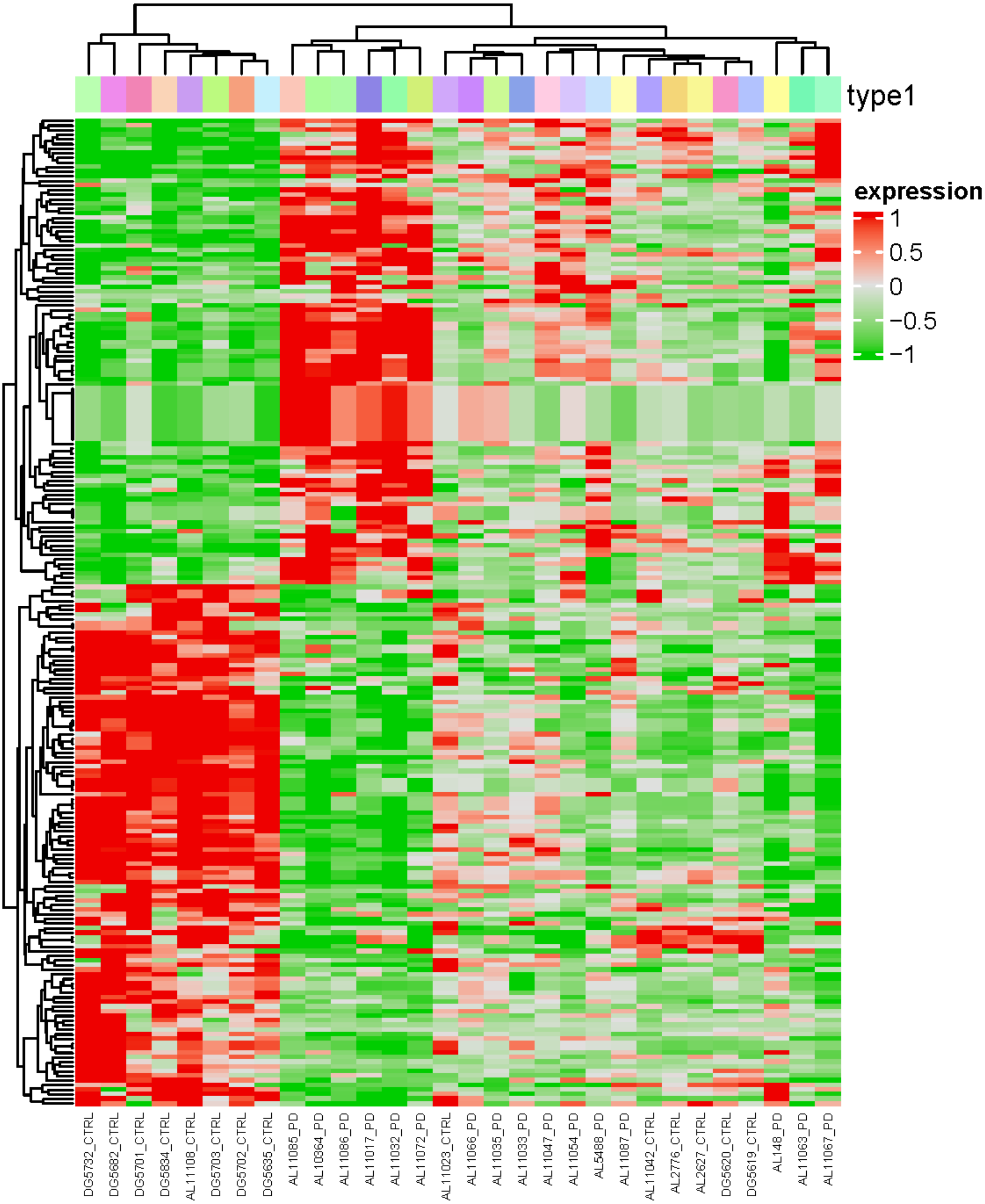

Figure S1. miRNA profiling. Heatmap of the unsupervised hierarchical clustering analysis on the expression profiles of the of differentially miRNAs (padj ≤0.05 and |FC| ≥1.5) in Parkinson's disease patients compared to healthy controls. Expression values lower or higher than the median are shown in green or red, respectively. Red: over-expressed miRNAs; green: downexpressed miRNAs.
