# Supplementary material for: Role and Dysregulation of miRNA in Patients with Parkinson’s Disease
Source: Int J Mol Sci. 2022 Dec 31;24(1):712. doi: 10.3390/ijms24010712 (PMC9820759; doi:10.3390/ijms24010712)
Supplement: Supplementary file 1 [file ijms-24-00712-s001.zip › Supplementary_Table_S8.pdf]

Table S8. Summary of the clinical-demographic characteristics of PD patients, along with main comorbidities and specific drug(s) taken.

N.	Sex	Age (years)	Disease duration	Phenotype (dominant side)	Psycho-cognitive status	Sleep disorder	Main comorbidities	Total daily levodopa equivalent dose (mg) [PMID: 21069833, PMID: 32258239]
1	M	71	6 years	Akinetic-rigid (right>left side)	Mild cognitive impairment	Mild-moderate obstructive sleep apnea syndrome	Chronic subcortical vascular disease, type 2 diabetes, arterial hypertension, atrial fibrillation	400
2	M	62	10 years	Tremor dominant (R > L)	Persistent depressive disorder with anxiety	Moderate obstructive sleep apnea syndrome	Chronic subcortical vascular disease, bilateral carotid stenosis, type 2 diabetes, dyslipidemia, obesity	705
3	F	73	2 years	Akinetic-rigid (bilateral)	Very mild cognitive impairment	None	Arterial hypertension, hypothyroidism, bilateral sensorineural hearing loss	0
4	F	62	7 years	Tremor dominant (bilateral)	Memory deficit, anxiety disorder	None	Mild chronic subcortical vascular disease, left carotid stenosis, obesity	0
5	M	68	10 years	Akinetic-rigid (L>R)	Depressive disorder	None	Left carotid stenosis, type 2 diabetes, dyslipidemia, bilateral sensorineural hearing loss	400
6	F	71	5 years	Akinetic-rigid (bilateral)	Normal	None	Arterial hypertension	800
7	M	61	1 year	Mixed (R>L)	Normal	None	Dyslipidemia	26
8	M	68	5 years	Akinetic-rigid (bilateral)	Mild memory deficit	None	Arterial hypertension, bilateral sensorineural hearing loss (R>L), benign prostatic hyperplasia	550
9	M	71	2 years	Mixed, > akinetic/rigid (R>L)	Mild cognitive impairment	None	Atrial fibrillation, chronic coronary artery disease	300
10	M	61	2 years	Akinetic/rigid (R>L)	Normal	Periodic limb movements during sleep	Arterial hypertension, obesity, bilateral sensorineural hearing loss	68

11	M	63	6 years	Mixed (L>R)	Normal	Obstructive sleep apnea syndrome	Arterial hypertension, obesity, hypothyroidism, bilateral sensorineural hearing loss (L>R)	720
12	F	66	15 years	Mixed (L>R)	Persistent depressive disorder	Insomnia	Dyslipidemia	585
13	F	72	9 years	Akinetic-rigid (R>L)	Normal	None	Arterial hypertension	1,050
14	F	69	4 years	Akinetic-rigid (R>L)	Mild cognitive impairment	None	Lacunar cerebrovascular disease, multiple disk protrusions, osteoporosis, psoriatic arthritis	250
15	M	74	2 years	Akinetic-rigid (bilateral)	Severe dementia	REM-sleep behavior disorder; moderate obstructive sleep apnea syndrome	Chronic subcortical vascular disease, arterial hypertension	1,200
16	M	76	6 years	Mixed (R>L)	Mild dementia	REM-sleep behavior disorder; obstructive sleep apnea syndrome	Chronic cortical-subcortical vascular diseases, bilateral carotid stenosis, chronic obstructive pulmonary disease, non-Hodgkin lymphoma, bilateral sensorineural hearing loss	615
